# Reducing the gap between science and clinic: lessons from academia and professional practice - part B: traditional vocal therapy techniques and modern electrostimulation and photobiomodulation techniques applied to vocal rehabilitation

**DOI:** 10.1590/2317-1782/20212021241en

**Published:** 2022-08-22

**Authors:** Mara Behlau, Anna Alice Almeida, Geová Amorim, Patrícia Balata, Sávio Bastos, Mauriceia Cassol, Ana Carolina Constantini, Claudia Eckley, Marina Englert, Ana Cristina Cortes Gama, Ingrid Gielow, Bruno Guimarães, Livia Ribeiro Lima, Leonardo Lopes, Glaucya Madazio, Felipe Moreti, Vanessa Mouffron, Katia Nemr, Priscila Oliveira, Marina Padovani, Vanessa Veis Ribeiro, Kelly Silverio, Thays Vaiano, Rosiane Yamasaki

**Affiliations:** 1 Centro de Estudos da Voz – CEV - São Paulo (SP), Brasil.; 2 Escola Paulista de Medicina, Universidade Federal de São Paulo – UNIFESP - São Paulo (SP), Brasil.; 3 Universidade Federal da Paraíba – UFPB - João Pessoa (PB), Brasil.; 4 Universidade Federal de Alagoas – UFAL - Maceió (AL), Brasil.; 5 Apta Comunicação, Recife (PE), Brasil.; 6 Universidade Federal de Pernambuco – UFPE - Recife (PE), Brasil.; 7 Centro de Fotobiomodulação e Saúde – CFOTOBIOS - Belém (PA), Brasil.; 8 Universidade Federal de Ciências da Saúde de Porto Alegre – UFCSPA - Porto Alegre (RS), Brasil.; 9 Universidade Estadual de Campinas – UNICAMP - Campinas (SP), Brasil.; 10 Faculdade de Ciências Médicas da Santa Casa de São Paulo – FCMSCSP - São Paulo (SP), Brasil.; 11 Universidade Federal de Minas Gerais – UFMG - Belo Horizonte (MG), Brasil.; 12 Clínica Bruno Guimarães Serviços de Fonoaudiologia e Fisioterapia, Fortaleza (CE), Brasil.; 13 Centro Universitário da Faculdade de Medicina do ABC – FMABC - Santo André (SP), Brasil.; 14 Complexo Hospitalar Municipal de São Bernardo do Campo – CHMSBC - São Bernardo do Campo (SP), Brasil.; 15 Universidade de São Paulo – USP - São Paulo (SP), Brasil.; 16 Faculdade de Odontologia de Bauru, Universidade de São Paulo – USP - Bauru (SP), Brasil.

**Keywords:** Voice, Voice Disorder, Voice Training, Therapy, Vocal Rehabilitation

## Abstract

This text is the continuation of the XVIII SBFa Congress publication. In part “A” we presented the analyses on clinical vocal evaluation. Part “B” focuses on vocal rehabilitation: 4. Traditional techniques of vocal therapy; 5. Modern techniques of electrostimulation and photobiomodulation applied to vocal rehabilitation. The numerous studies on the various programs, methods, and techniques of traditional rehabilitation techniques, and many with high quality of evidence, allow us to consider such procedures relatively well described, safe, and with known effects, accounting for the treatment of various vocal disorders. The scientific evidence with traditional techniques is recognized worldwide. New fronts of evolution, with electrostimulation or photobiomodulation used to handle voice problems, seem to be promising as coadjutant approaches. There are more studies on electrostimulation in vocal rehabilitation than with photobiomodulation; however, scientific evidence for these two modern techniques is still limited. Knowledge and caution are required for the application of either technique.

## INTRODUCTION

The Scientific Session in the Merit Room Mara Behlau, held on December 14, 2020, as one of the activities of the XXVIII Brazilian Congress of Speech-Language Pathology, organized by the Brazilian Society of Speech-Language Pathology (*Sociedade Brasileira de Fonoaudiologia* – SBFa), was consolidated in two publications. In part “A”^(1)^ of this article, the main scientific data were presented, comparing them with the Brazilian clinical reality regarding the first three aspects discussed, namely: 1. Auditory-perceptual judgement of vocal quality; 2. Acoustic analysis of the vocal signal; 3. Voice self-assessment. In this second publication, part “B”, aspects related to vocal rehabilitation are explored, presented in two topics: 4. Traditional techniques of vocal therapy; 5. Modern techniques of electrostimulation and photobiomodulation applied to vocal rehabilitation.

Vocal rehabilitation or speech-language therapy for voice is understood to be a dynamic and non-linear process to develop a better balance of structures and processes that account for vocal functionality, reducing a voice handicap or improving the aesthetic qualities of an emission^(2)^. Historically, it is a combination of arts and science, with the first voice exercises coming from teaching singing. From the 1950s onwards, there was a philosophical repositioning of vocal rehabilitation, with the search for scientific foundations for the proposed techniques, based on the seminal works of Emil Froeschels^(3,4)^. The evolution of knowledge took place in several domains, but, in general, the contributions to the vocal rehabilitation processes involve three aspects commonly present in voice therapy, distributed in variable doses according to the case^(2)^: 1. Behavioral changes, 2. Muscle adjustments and/or 3. Issues of vocal self-image,

Speech-language pathology rehabilitation acts on vocal functionality and a good part of this action is due to the techniques used. In the present text, the main focus is precisely on vocal rehabilitation methods and techniques, as they occupy a central space in speech-language pathology rehabilitation, as they allow the individual to experiment with new ways of producing the voice, with more efficient muscular adjustments, favoring the experience of produce a more harmonic voice with less effort after a few minutes of performing selected exercises. The vocal rehabilitation process is didactically presented in two components: 1. Direct vocal therapy, directed to the vocal technique itself; 2. Indirect vocal therapy, which includes approaches to deal with cognitive, behavioral, and environmental aspects, such as stress management and problem coping, relaxation techniques, vocal counseling, and environmental aspects such as a microphone, room acoustics, especially in professional voices. Although we recognize the importance of both aspects, we chose direct vocal therapy as a topic of discussion to reduce the gap between science and clinic, because when analyzing scientific evidence, the main ones focus on direct approaches^(5)^, analyzed in this publication.

All applied science and, in general, the area of health, presents two movements for the advancement of knowledge: the verification of the so-called traditional approaches, often used for a long time and developed intuitively, and the proposition of innovative interventions, sometimes already used in other fields of knowledge. Technological advances and scientific research have been modifying the clinical routine of the Speech-Language Pathologist, whether regarding the evaluation of the case, as highlighted in part “A” of this contribution^(6)^, but also the administration of therapy procedures. Based on this statement, we present the main data of traditional vocal rehabilitation techniques, followed by consideration of two approaches recently suggested as adjuvants and used in the vocal clinic, electrostimulation and photobiomodulation.

## TRADITIONAL VOCAL TECHNIQUES IN VOCAL REHABILITATION

It is a challenge for the voice specialist to apply an efficient treatment using the best available scientific evidence. Combining the results of scientific research with clinical decisions involves several paths, ranging from the analysis and interpretation of the results of scientific research to the formulation of evidence and guidelines for clinical practice^(7)^. As explained in part “A” of this contribution^(6)^, approaches such as evidence-based practice^(8)^ incorporate the conscious and thoughtful use of the best research evidence in making clinical decisions. This may seem to limit the available options to use only proven techniques and methods. Although this seems to be the best attitude, the clinic often requires the use of procedures that still lack evidence. In these cases, transparency in the relationship with the patient and the search for, at least, clinical evidence is essential.

Based on the above, we present the main scientific evidence and clinical decisions of the most researched methods and techniques of vocal rehabilitation in the voice area. They are: Lee Silverman Method, LSVT®, Vocal Function Exercises (VFE); Resonance Method, Comprehensive Vocal Rehabilitation Program - CVRP; Accent Method; Vocal Therapy for the Elderly – VTE; Phonation Resistance Training Exercises – PhoRTE; Proprioceptive-Elastic Method – PROEL; Laryngeal Massage Technique; Semi-occluded Vocal Tract Exercises – SOVTE and Basal Sound Technique (vocal fry).

The **Lee Silverman Method, LSVT**® presents the exclusive focus on increasing voice intensity as essential concepts, with multiple repetitions in maximum phonation time and with voice frequency variation; it is applied intensively, with four sessions per week for four weeks, totaling 16 sessions^(9-11)^. Its results are based on a systematic review of the literature^(12)^, with a meta-analysis of nine randomized clinical trials^(9-11,13-17)^ that evidence this method as a treatment that offers improved communication in patients with idiopathic Parkinson's disease, both in the form of telecare and in the face-to-face format, promoting improvement in vocal intensity and positive impacts on quality of life. This method is the most studied in vocal rehabilitation, with the highest level of proven scientific evidence, widely used in several parts of the world; however, it requires a specific certification for its administration.

The **Vocal Function Exercises Method - VFE** is a physiological approach to the treatment of voice disorders based on the development of a physiological balance between the breathing, phonation, and resonance subsystems to strengthen the intrinsic muscles of the larynx and increase the resonance of the vocal tract^(18)^. A systematic review containing 21 clinical studies^(19)^, eight of which were randomized clinical trials^(18,20-26)^ presents a moderate to a strong level of evidence for VFE as an efficient treatment method to improve the vocal function of individuals with different conditions of dysphonia, presbyphonia, and for voice professionals^(19)^.

The **Resonance Method** has different names and can be considered a vocal method or technique: 1. Method: Resonant Voice Therapy^(27)^, Lessac-Madsen Resonant Voice Therapy - LMRVT^(28)^, Resonance Therapy^(29)^; and, 2. Techniques: Humming^(30)^, Nasal Sounds Technique31, and Y-Buzz^(31)^. In Brazil, it is mostly used as a vocal technique^(32)^. Its application, either as a method or as a vocal technique, aims to improve vocal production, minimizing the impact between the vocal folds and, consequently, injuries from speech trauma^(27,33)^. According to a systematic review that included nine clinical trials^(34)^, two of which were randomized clinical trials^(27,33)^, this method has a moderate level of evidence for behavioral dysphonia, with improved voice quality and phonatory efficiency^(34)^.

The **Comprehensive Vocal Rehabilitation Program** - **CVRP** is a vocal rehabilitation method proposed by a Brazilian group^(35)^, which focuses on five aspects: body-voice, glottal source, resonance, pneumophonoarticulatory coordination, and communicative attitude. The results of a randomized clinical study^(26)^ evidence the effectiveness of CVRP, which promotes positive results in vocal quality, laryngeal function, and improvement in the quality of life of patients with behavioral dysphonia.

The main objective of the **Accent Method** is to obtain better control of speech and voice production, through adequate pulmonary support associated with rhythmic emissions of vowels and phrases and body movements, which restore the vibration symmetry of the vocal folds and reduce glottic muscle effort^(36)^. Its holistic approach was proven through a randomized clinical trial^(37)^ that showed improvement in vocal quality in cases of hyper and hypofunctional dysphonia.

The **Vocal Therapy for the Elderly – VTE** program was developed in Brazil to specifically treat presbyphonia^(38)^. The results of the randomized clinical trial^(38)^ prove the effectiveness of VTE in the vocal treatment of the elderly, promoting improvements in quality of life and voice quality immediately after treatment and a one-month follow-up. The benefits obtained by VTE in the intensive or conventional format are similar, differing only in the decrease in vocal fold bowing, which was more evident in the intensive format^(38)^.

The **Phonation Resistance Training Exercises - PhoRTE**, is a method of vocal rehabilitation for elderly people with presbyphonia^(23)^ adapted from the LSVT®^(9)^ and which focuses on increasing vocal intensity and voice projection. A randomized clinical trial^(23)^ showed that PhoRTE improves the quality of life and self-perception of phonatory effort in presbyphonic individuals.

The **Proprioceptive-Elastic Method - PROEL** is a holistic Italian method of vocal rehabilitation for functional and organic dysphonias^(39)^. This method aims to rebalance the phonatory system, eliminating muscle tension and seeking greater elasticity in the body. Less muscle tension in the body produces greater elasticity of the phonatory system, leading to a maximum ecological state. Research with a before-after intervention design showed an improvement in the vocal quality of patients with functional dysphonia^(39)^ and a quasi-experimental study showed an improvement in the voice quality of patients undergoing partial horizontal laryngectomy^(40)^.

The **Laryngeal Massage Technique** is one of the vocal clinic options with the highest level of scientific evidence. There are variations in its application, which can be performed with intense manipulation via the anterior region of the neck and associated vocalizations^(41)^ or by posterior access, with gentle action of both hands, without vocal emission during manipulation^(42)^. A systematic review^(43)^ that analyzed two randomized clinical trials^(44,45)^ and two before-after intervention studies^(46,47)^ indicated the efficiency of this approach applied to behavioral dysphonia, through muscle balance acquired with the relaxation of the paralaryngeal muscles and flexibility of movement vertical position of the larynx, with a decrease in the intensity of body pain.

The **Semi-Occluded Vocal Tract Exercises Technique - SOVTE** includes a wide range of exercises with closed vowels, voiced fricative sounds, tongue, lips, or lingual-labial vibration, in addition to vibratory exercises with various artificial extensions of the vocal tract, that is, tubes of resonance, flexible or rigid, wide, and narrow straws, for sound emission in the air or the water. Considering primarily the SOVTEs that used tubes or straws, the most relevant scientific research is the systematic review of SOVTE in the singing voice^(48)^, which analyzed six eligible articles^(49-55)^. According to this study^(48)^, exercises with a resonance tube immersed in water, flexible tube, and straws promote positive effects on the vocal quality of singers, with more comfortable, more projected, and more economical emission. Several other contributions^(51,56-60)^, including two randomized clinical trials^(61,62)^ showed that SOVTE can be indicated for cases of vocal hyperfunction, behavioral dysphonia, and for vocal conditioning, helping in source-filter tuning and more efficient glottal adduction. As for the vibrating sound techniques, considered facilitating sounds, the greatest evidence is obtained in two randomized clinical trials^(62,63)^. The clinical application of this technique produces several effects and aims at a balanced and comfortable vocal emission, better glottic closure and greater number of harmonics. Another technique that is part of SOVTE is glottal firmness; although some studies have been performed with this technique, mainly comparing it with other SOVTE^(50,58,64)^, only one randomized clinical trial^(65)^ has been published. Glottic firmness promotes better glottic adduction, increased mucosal vibration amplitude, and vocal tract enlargement.

The level of evidence for the **Basal Sound Technique** is from a randomized clinical trial. The basal sound is a facilitating sound emitted with greater contraction of the thyroarytenoid muscle, capable of generating the deactivation of the usual muscle adjustment, stimulating mucomodulatory movement, and favoring glottic adduction. Although relatively difficult to perform, as it requires the production of an unusual sound in communication, the vocal fry, its effect commonly occurs producing better vocal quality and easier emission.

With all these results and evidence, the decision of the Speech-Language Pathologist specialist in voice in choosing the best procedure, method, or technique should be based on two main axes: 1. With regard to research, the question that needs to be answered is: how to elaborate the best research design to develop scientific knowledge applied to speech-language rehabilitation?; 2. on the vocal clinic axis – which clinical procedure presents the greatest evidence of scientific research? There is important evidence in traditional methods of vocal rehabilitation, some with more holistic proposals and others more specific for certain vocal conditions. Evidence of the use of vocal rehabilitation in pediatric dysphonia is lacking, although the possibility of long-term vocal risks is recognized, especially in girls^(66)^.

## MODERN TECHNIQUES OF ELECTROSTIMULATION AND PHOTOBIOMODULATION APPLIED TO VOCAL REHABILITATION

### Electrostimulation in vocal rehabilitation

Several electrical currents can be used as a therapeutic resource with different objectives, whether for pain management, progress in vascularization, tissue healing, activation of cellular metabolism, improvement of proprioception, relaxation, drainage, or muscle contraction. Among the numerous existing currents, the most used in the voice area are TENS (Transcutaneous Electrical Nerve Stimulation) and FES (Functional Electrical Stimulation); in the international literature, this last current is sometimes called NMES (Neuromuscular Electrical Stimulation), but there are several discussions about it due to the confusing nomenclature brought from the area in which we support our knowledge, which is Physiotherapy.

The fundamental parameters in electrotherapy that define the type of electric current are frequency, pulse duration, and intensity, in addition to the waveform. The combination of these four parameters will define the type of current and several different types of currents and, therefore, have different effects on the tissues.

Specifically, about TENS, professionals from the speech-language pathology clinic and researchers in ​​electrostimulation agree and realize that there is confusion regarding the name of this current and its variations. The name TENS is not enough to show its combination of parameters. Thus, it would be more appropriate to identify the procedure using the first name “TENS” plus the second name, such as: “TENS ACUPUNCTURE - in low-frequency modulation”; “TENS CONVENTIONAL – in high-frequency modulation”; “TENS BRIEF INTENSE”; “TENS BURST”; “TENS VIF - Variation of intensity and frequency”.

Electrostimulation in the voice area has been used in Brazil since 1986, applied to cases of laryngeal paralysis in the vocal clinic, using TENS BURST, to stimulate muscle contractions in the larynx. Soon after, TENS was applied in behavioral dysphonia, using high-frequency TENS BRIEF INTENSE, to promote laryngeal relaxation. With the advancement of knowledge, high-frequency TENS CONVENTIONAL was applied for cases of dysphonia accompanied by acute muscle tension and low-frequency TENS ACUPUNCTURE for cases of dysphonia with chronic muscle tension or chronic laryngeal pain. The first Brazilian publication on electrostimulation^(67)^ was a case report of a patient with spastic dysphonia in adduction, in which TENS was applied associated with breathing and phonation exercises, to achieve muscle relaxation, obtaining positive results in the voice and larynx up to 60 days after starting treatment. However, research on electrical stimulation in Brazil began only in 2008, with a group of researchers with physical therapists and Speech-Language Pathologists applying low-frequency TENS Acupuncture in women with behavioral dysphonia^(68)^. Therefore, there is still much to be investigated and proven in this area.

In the case of behavioral dysphonia, some studies were carried out demonstrating two different ways of applying TENS. “TENS Acupuncture - in low-frequency modulation”, but with high intensity at the motor threshold^(45,68-72)^, in which two electrodes from the same channel of the current generating device are distributed ipsilaterally: one fixed in the region of the trapezius muscle – descending fibers and the other in the submandibular region (and the same distribution is repeated on the opposite side), with a wider stimulation field, but with less deep stimulation. In this current configuration, the individual receives the stimulus in the supine position and there is no emission, or any voice exercise associated with the current, during electrostimulation, because the stimulation received is strong, causing a strong shock in the larynx. The other form of current research and widespread in the vocal clinic is “TENS Acupuncture - in low-frequency modulation”, but with low intensity, in the sensorimotor threshold^(73-77)^. In this type of stimulation, two electrodes from the same channel of the current-generating device are placed bilaterally on the thyroid cartilage plate, stimulating a smaller field, in which the stimulation is more concentrated and deeper in the larynx region. In this type of current, the association of vocal exercises is recommended, and the intensity of electrostimulation is light.

Clinical experience and research agree and demonstrate that both forms of TENS promote analgesia, decrease phonation effort, lead to muscle balance, with the improvement of laryngeal and vocal symptoms, as well as improvement of vocal quality, especially regarding vocal tension^(45,69-72,74-78)^. Such effects were verified by comparing the moments before and after the intervention with electrostimulation, through self-assessment protocols, auditory judgement, and acoustic assessment, either in an immediate effect study or in an intervention for 12 sessions.

Although the clinic and the research have several converging points, there is still little scientific evidence about electrical stimulation in the rehabilitation of patients with dysphonia. The literature reports 13 studies, with different levels of evidence, two of which are systematic reviews^(78,79)^, seven randomized clinical trials^(69-71,74,78,79)^ three quasi-experimental studies^(75,76,80)^ and a pre-post effect study^(68)^. Most studies address behavioral dysphonia, and this is the greatest contribution of Brazilian research. However, some weak points deserve attention: long time between completion of the research and publication; small samples; laryngeal diagnosis as an inclusion criterion and the presence of other muscular and vocal signs that make the sample less homogeneous; difficulty in performing a more objective assessment of the laryngeal image and difficulty in following up treated cases.

On the other hand, the clinical experience of colleagues who have used such procedures reveals that working with “TENS Acupuncture - in low-frequency modulation”, with low intensity in the sensorimotor threshold, with stimulation field in the larynx, can regulate the strength of muscle, functioning as biofeedback regarding the vocal intensity used during the voice exercise, improving proprioception, leading to laryngeal balance more quickly. However, the clinic has some weaknesses, recognized by the specialists themselves, such as the difficulty of showing their results in the literature, as well as the difficulty of properly documenting the treated cases, applying knowledge to a greater number of patients. Thus, decision-making is not always based on scientific evidence in the area, as clinical experience is highly considered since research does not bring such rapid results in the literature for the consumption of clinical Speech-Language Pathologists.

Some points where research and clinic have difficulties in converging are about the necessary control in scientific research about the environment, sample, applicability of the intervention and outcomes to be evaluated necessarily equal to validate the results, which is not possible to be implemented in the clinical care. For scientific studies, the current applied in interventions with electrostimulation must remain the same, even if the needs and muscle characteristics of individuals are different. On the other hand, electrostimulation in the clinic is dynamic and customized for each patient, with the clinician being autonomous in choosing the parameters of the current to be applied.

The current forms have specific indications and purposes to be considered when prescribing. The effectiveness of electrostimulation depends much more on professional knowledge/competence in decision making and the application of the technique. However, researchers and clinicians agree that there is a need for more studies with a higher level of scientific evidence and to answer the various questions raised by clinical practice. It is important to consider future research with FES, AUSSI, and RUSSA, not only in behavioral dysphonia but also in organic ones. There is research with FES, however, the level of evidence is very low^(81-83)^.

Given the above, clinicians and researchers agree that TENS can be applied alone in cases of dysphonia accompanied by muscle tension and pain. If TENS ACUPUNCTURE is applied in the cervical region, with strong intensity in the motor threshold, exercises or any type of phonation should not be associated. When choosing TENS CONVENTIONAL, with electrodes in the laryngeal region and light stimuli, in which muscle tension is not accompanied by pain, the current associated with traditional vocal techniques can be applied. Both forms of application are considered adjuncts in the therapeutic process.

Multiparametric measurements may help to better understand the results of interventions with electrical stimulation, as well as assessments involving dosimetry. The interaction between research and clinic is a fundamental aspect to be considered in the resolution of the questions raised by both for the scientific advance in the voice area.

### Photobiomodulation in vocal rehabilitation

Photobiomodulation therapy consists of a therapeutic modality in which the irradiation of light in the visible and/or near-infrared spectrum promotes biophysical and biochemical changes in the body, resulting in biological responses such as modulation of inflammation, reduction of edema, pain, and improvement of tissue regeneration^(84)^. This type of therapy has been known for more than five decades but has not yet gained wide acceptance, mainly because molecular, cellular, and tissue mechanisms of action are not well accepted^(85)^.

Among the various light sources that can be used for this purpose, in Speech-Language Pathology, the low power LASER (Light Amplification by Stimulated Emission of Irradiation) and the LED (Light Emitting Diodes) stand out, especially the wavelengths in the range of red (635–700 nanometers) and near-infrared (808-1100 nanometers). The effect of LASER used in photobiomodulation therapy is neither thermal nor ablative. For this reason, the equipment used is those registered with the Brazilian Health Regulatory Agency (*Agência Nacional de Vigilância Sanitária* – ANVISA), which gives security to the clinician as they have been verified and approved. The LASER used in the vocal clinic is of low power, without bionegative effects on the biological tissue, acting mainly in stimulating the organism's physiology. The therapeutic effects of phototherapy stem from the absorption of photons by chromophores, which are sets of atoms or parts of a molecule capable of absorbing light in the ultraviolet or visible spectrum region and are also responsible for the color of a molecule^(86)^.

Although the cellular mechanisms associated with photobiomodulation are not yet fully understood^(85)^, the most accepted theory to date postulates that red and infrared absorption occurs in the mitochondria, more precisely in the respiratory chain, favoring the production of adenosine triphosphate (ATP), the regulation of intracellular pH, activation of antioxidant mechanisms and recovery of cellular homeostasis^(87,88)^.

The use of these therapeutic resources in ​​the voice area is still poorly explored and, although it has become popular in the last decade, it is based on evidence from other health specialties, such as physiotherapy, dentistry, cardiology, and sports medicine. In cases of edema and inflammation, for example, light therapy improves local microcirculation, angiogenesis, inhibits chemical mediators, and activates defense cells and antioxidant enzymes^(89)^. In tissue regeneration, in turn, evidence demonstrates effectiveness both in superficial lesions and in deeper tissues^(90)^, proving to be efficient in the proliferation of fibroblasts, in the synthesis and organization of collagen, and the induction of neovascularization^(91,92)^. In sports medicine, the effects of photobiomodulation on the muscular system are increasingly robust, with emphasis on the increase in resistance to fatigue, strength, and recovery speed of athletes^(93-96)^ as well as its effectiveness in cervical and muscle pain relief^(97)^.

Overall, effects such as edema reduction, inflammation modulation, improved muscle performance/relaxation, and tissue recovery were analyzed in a systematic review that showed positive results in 13 of 16 studies, despite heterogeneity and methodological issues^(95)^. The application of photobiomodulation therapy associated with exercise in healthy subjects showed improvement in muscle performance and reduction of fatigue in a recent systematic review with meta-analysis, comparing the results of 28 studies against placebo^(95)^. Finally, an integrative review showed that Brazilians have stood out in publications on photobiomodulation in the health area and, in general, low-intensity LASER acts on muscle performance, reducing fatigue, increasing strength, muscle resistance, and improving chemical markers; the methodological diversity of the studies made it difficult to identify effective parameters to obtain results^(98)^.

In vocal science, a pioneering study on the use of photobiomodulation for the treatment of intubation-induced laryngopharyngeal reflux laryngitis in rats showed that laser benefited laryngeal tissue repair in irradiated animals when compared to non-irradiated animals. According to the authors, the results indicate that the use of this resource can be beneficial for the population with voice disorders, including those with tissue alterations in the lamina propria^(99)^. The LED was tested as a possibility of attenuating the symptoms of vocal fatigue^(100)^, the red light was effective to improve markers related to acoustics, aerodynamics, and self-perception of healthy individuals submitted to a vocal effort task. The findings indicate that photobiomodulation can be a promising resource for the reduction of vocal fatigue. The ideal dosimetric parameters, the combination or not of photobiomodulation with vocal exercises, and the ideal moment of application should be determined. Finally, the use of photobiomodulation in human vocal folds, *in vitro* ^(101)^ and *vivo* ^(102)^ in rabbit vocal folds, revealed that the red wavelength increased the proliferation and migration of vocal fold epithelium cells, pointing to a future possibility of using this resource in the healing of laryngeal lesions.

Although the available data are still preliminary and recent, phototherapy is a simple treatment that can bring benefits to the voice area, especially in healthy individuals with a focus on muscle training. However, its use must be controlled by qualified professionals who can apply adequate doses, at the right time, in the right location to achieve the desired effect. As observed in electrostimulation, the application of photobiomodulation must be based on a complete knowledge of the general clinical conditions and local application, with special attention to the head and neck region.

## COMMENTS

The literature shows many studies in the ​​voice area with the most varied vocal techniques, with different designs and levels of evidence. Research has advanced, there are randomized clinical trials with specific techniques and systematic reviews. Much attention has been paid to understanding the effects of traditional voice therapy techniques, and we can say that the past decade was the decade of studies related to proving the effects of voice therapy^(6)^. The results of scientific research allow us to recognize that traditional approaches account for the variety of vocal cases, are safe, have relatively defined steps, and many of them are used worldwide, such as the Resonance Method^(27-34)^. It is also recognized that traditional approaches to vocal rehabilitation have an effect not only on the voice but also on competence in communication and emotional aspects of patients^(6)^. Both specific methods, such as LSVT, for individuals with Parkinson's Disease^(9-11)^, and holistic methods, such as PROEL^(39,40)^ produce immediate and short-term positive effects. The greatest concentration of quality evidence on the effects of speech-language intervention is concentrated on traditional rehabilitation approaches.

A survey of the entire collection of the Journal of Voice - JoV positions Brazil as the second country that most contributed to publications on therapy, second only to the United States of America^(6)^. This analysis of JoV publications over the decades clearly showed the evolution of Speech-Language Pathologists as researchers, through studies with scientific writing and better definition of terms, better methodological design, clearer definition of variables, adequate selection of outcomes, a better description of participants, and positive effects, to varying degrees, of therapy techniques, methods, and programs. It is interesting to note that 49 different approaches^(6)^ were identified, used alone or in combination, which allows considering that the methods, techniques, or programs approaches have some common active components, which should be better understood in a taxonomy proposal for voice therapy. Recently, a proposal of this nature^(103)^ grouped the instruments of direct intervention into 5 categories, according to the subsystem primarily involved in the execution of the exercises, namely: 1. Auditory; 2. Vocal function; 3. Musculoskeletal; 4. Respiratory; and 5. Somatosensory. This taxonomy of therapeutic programs, using standard terminology and a framework to help systematic research, aims to reduce the “black box” phenomenon in voice therapy, which makes it difficult to understand the active ingredients of a rehabilitation program, impairing the development of the clinical and scientific specialty.

As for the modern techniques recently used in voice therapy, electrostimulation has been presented as an interesting therapeutic option when associated with traditional techniques in behavioral conditions, with laryngeal hyperfunction or in conditions of hypofunction, of neurological or functional causes, demanding different clinical reasoning in the selection of this feature. For both indications, it is worth mentioning that it is a strategy that, in addition to favoring an increase in muscle strength or helping to reduce excessive muscle contractions, can favor the creation of feedback that maximizes muscle performance after voluntary muscle contraction exercises^(104,105)^. This possibility can facilitate the organization of new laryngeal motor engrams, by providing tactile-proprioceptive feedback, facilitating repetition, or breaking of a pattern that needs to be modified.

More recently, electrotherapy has been discussed as an important modulating agent during the execution of volitional motor action, influencing functional performance^(106)^. Therefore, the use of this resource can promote sensorimotor integration, in which the sensory pathways are sensitized by the peripheral proprioceptive feedback caused by the movement of the segment, momentarily activating the sensorimotor cortex^(107)^. In this way, a focus on sensory modulation can also be considered, bringing new possibilities to be researched in conditions of hyper or hyposensitivity, such as refractory chronic cough, for example.

Studies analyzed in an integrative review^(108)^ point to benefits with electrostimulation in the rehabilitation of patients in the speech-language pathology clinic, however, there is methodological diversity, making it difficult to compare the findings. It is suggested selection of more homogeneous samples, with a detailed methodological description of the dosimetry used and not only immediate effects. The advances observed in the use of electrotherapy associated with traditional speech-language pathology techniques do not dispense with the careful observation of contraindications, especially in the region to be applied, the cervical, richly vascularized, with the presence of the carotid sinus, and innervated by part of the cranial nerves, in addition to other aspects, such as the use of other electrical devices and epilepsy, for example.

Given what has been exposed, it is important to use evaluation methods in clinical routine and in research to verify patients' muscle conditions, seeking greater effectiveness in the application of TENS. An analog scale for musculoskeletal pain that assesses the perilaryngeal region at rest and during phonation, the Laryngeal Palpatory Scale^(109)^ can be used. A recent systematic review^(79)^ analyzed 8 out of 100 articles that met the inclusion criteria, from two respected laboratories (Brazil and Iran) with 87.5% of the studies showing effective results after the intervention, with reduction of pain in the areas of the larynx, neck, shoulders, back, and masseter. The studies also showed improvement in the auditory-perceptual judgement of vocal quality and reduction in sensations of pain, swelling in the throat, and effort to speak. The physiotherapy literature, which has already used this approach in many conditions and for a long time, highlights the need for knowledge of the physiological and methodological characteristics for its application, both in healthy musculature and in altered musculature, so that the optimization of the use of the technique can be conducted effectively and safely in clinical practice and for research purposes^(110)^.

We emphasize the need for caution in the application of electrostimulation and the need to know the anatomy in the cervical region to ensure a safe and responsible practice. Speech-language pathologists traditionally work in health teams, with good relationships with all members of the individual’s care team, who can confirm the application of TENS when necessary. This responsible practice for the application of TENS, or any other form of electrostimulation, is essential for the professional who intends to use this resource in the head and neck region.

Finally, as for modern photobiomodulation therapy, this is a non-invasive technique applied by many disciplines. Specifically, regarding vocal issues, photobiomodulation applied to the laryngeal region aims to improve vocal quality; however, there are still no scientific studies that prove positive evidence of its use. It should be noted that, despite its potential, the use of phototherapy should only be indicated as an adjuvant therapeutic resource, informing the client/patient that its effects are not yet scientifically proven in the voice area. Concerning the application technique, it is essential to proceed with adequate palpation of the laryngeal region and its respective structures, to obtain safety and precision in the area to be irradiated.

Some contraindications include history of carcinoma and irradiation of the neck region^(111)^. The cervical region is highly vascularized, and it is essential to know the anatomy and physiology of the patient before proposing this procedure, as well as the application techniques and their contraindications. Care should be taken with a previous medical diagnostic evaluation, with complementary tests, since conditions such as vocal fold hemorrhages, neoplasms, papilloma, or leukoplakia require caution in use, some of which are contraindications for the use of photobiomodulation. Special attention should also be given to the thyroid gland, located in the anterior region of the neck with an upper limit at the lower border of the thyroid cartilage. As the effect that this resource may have on glandular tissue is not yet known, it is suggested to investigate possible thyroid alterations, with greater emphasis on hyperthyroidism^(111)^. The investigation of the use of photosensitive drugs that can cause stains on the patient’s skin with the use of light also deserves attention.

Photobiomoduction has also been used as a treatment in patients with neurological diseases, such as Parkinson’s and stroke sequelae, to improve brain metabolism and neuronal regeneration^(110)^, as well as in oncology in the treatment of radiomucositis and xerostomia resulting from radio and chemotherapy. In this sense, it also plays a role in the treatment of patients with voice disorders and dysphagia associated with these diseases; however, caution is required in the use of photobiomodulation, especially in cancer patients, due to the potential effect of tumor reactivation^(112)^.

Photobiomodulation, by itself, is not able to reabsorb or eliminate lesions and also does not influence voice-damaging behaviors. Therefore, it is essential to obtain good results that the professional prioritizes clinical reasoning and not a therapeutic resource, in an isolated way.

The data presented show the dynamism in the voice area and the search for scientific studies to be reflected in conscious clinical practice. The main comments on the six aspects of the vocal clinic discussed in parts “A” and “B” of this text are presented in Chart 1.

**Chart 1:** Important points of the six aspects of the vocal clinic discussed: auditory-perceptual judgement, acoustic assessment, voice self-assessment, vocal techniques of vocal therapy, laryngeal electrical stimulation, and voice photobiomodulation

**Vocal clinic aspects**	**Important points**
Auditory-perceptual Judgement	• Auditory-perceptual judgement is considered the gold standard in the vocal clinic because the voice is a perceptual phenomenon.
	• Professional judgement requires specific training.
	• Reliability varies according to the type of stimulus, task, the experience of the evaluators, among others.
	• Roughness, breathiness, and strain are the three main vocal deviations.
	• GRBASI and CAPE-V scales are the most used.
	• GRBASI is simpler but less sensitive to less obvious changes in vocal quality.
	• CAPE-V requires more training but better identifies small increments with treatment
Acoustic Evaluation	• Essential documentation in the vocal clinic.
	• Quality of recording is crucial to its reliability.
	• Allows evaluation of source and filters contribution.
	• Provides vocal production information.
	• Public-use or low-cost programs are available.
	• Makes the voice more concrete, allowing visuo-vocal association.
	• It can be performed descriptively, regardless of the type of vocal signal, by spectrographic analysis.
	• It can be performed by extracting acoustic parameters depending or not on the extraction of the fundamental frequency.
	• Isolated parameters that depend on the extraction of the fundamental frequency have limited value.
	• Multiparametric indexes or cepstral measurements are more reliable and promising.
	• Moderate correlation with auditory-perceptual judgement.
Voice self-assessment	• Unquestionable contribution to the vocal assessment.
	• Explores the unique perspective of those living with a voice problem.
	• Offers data that cannot be estimated by other assessments.
	• Protocols developed in other languages require cultural and linguistic adaptation and validation with verification of psychometric properties for use in Portuguese.
	• Together with the auditory-perceptual judgement and the acoustic assessment, it forms an important triad of individual vocal assessment.
Traditional Vocal Therapy Techniques	• There are a variety of techniques, methods, and programs analyzed with studies of different designs and variable methodological quality.
	• The greatest evidence of scientific proof of vocal therapy was produced with these techniques.
	• Programs such as LSVT and VFE concentrate the largest number of studies.
	• Semi-occluded vocal tract exercises are varied, described a long time ago, and submitted to several scientific studies.
	• Brazil occupies an important space in the study of traditional vocal therapy techniques
Electrostimulation in Vocal Rehabilitation	• Used in other health sciences, it has shown interesting results in the vocal clinic.
	• It has already produced high-quality evidence of its effect in the treatment of certain dysphonias.
	• The type of current used has special consideration in clinical application.
	• Its different currents and parameter settings can allow sensory, motor, or both laryngeal focus.
	• It is seen as an adjunct treatment and not as a sole resource in cases of dysphonia
Photobiomodulation in Voice	• Used in other health sciences, it still needs to produce scientific evidence in the vocal clinic.
	• The choice of dosimetric parameters has special consideration in clinical application.
	• It is seen as an adjunct treatment and not as a sole resource in cases of dysphonia.
	• Can also be used for vocal conditioning

## CONCLUSION

Vocal rehabilitation is the responsibility of the Speech-Language Pathologist, Voice Specialist, who works to recover vocal functionality and obtain the best possible result according to the patient’s anatomy functional condition. The options for proposing a rehabilitation program are varied. Choosing wisely is an essential attitude for the improvement of speech-language therapy services and a positive perception of other health professionals, health insurance companies, and the lay public. Exactly this statement “Choosing Wisely” was the motto of a campaign created by the American Board of Internal Medicine – ABIM, in 2012, to reduce the use of low-value interventions for the patient, avoiding tests or unnecessary clinical practices^(113)^. This campaign, launched in 2012, is now joined by more than 70 health societies and more than one million clinical partners in the campaign. Avoiding unproven practices is a professional responsibility and is very present in recommendations in neurology, obstetrics, intensive care, geriatrics, pediatrics, nursing, and physiotherapy, among other disciplines.

Despite great clinical and scientific vitality, the ​​voice area still needs to advance in research to obtain better evidence. Evidence on traditional therapy techniques is available in the literature, much of it produced by Brazilian groups. Researchers need to have better training to propose a more robust design of experiments. Clinicians need the training to consume science consciously and critically and to make appropriate decisions. It is worth remembering that research results are often interpretive and obtained under ideal laboratory conditions. All these aspects must be considered. Reducing the gap between science, through research analysis, and the vocal clinic, which must indicate which problems deserve to be investigated, must be a shared responsibility by the professionals of a discipline.

Choosing wisely is honoring scientific research, which is necessary for the clinical advancement that we seek so much, in addition to reflecting respect for the patient who comes to us. An academic training supported by the best available knowledge and a conscious and science-compatible practice are important academic responsibilities of all institutions and clinicians who propose to teach their colleagues.
