# Supplementary material for: Reducing the gap between science and clinic: lessons from academia and professional practice - part B: traditional vocal therapy techniques and modern electrostimulation and photobiomodulation techniques applied to vocal rehabilitation
Source: Codas. 2022 Aug 22;34(5):e20210241. doi: 10.1590/2317-1782/20212021241en (PMC9886189; doi:10.1590/2317-1782/20212021241en)
Supplement: Translation

# Translation: Reduzindo o gap entre a ciência e a clínica: lições da academia e da prática profissional – parte B: técnicas tradicionais de terapia vocal e técnicas modernas de eletroestimulação e fotobiomodulação aplicadas à reabilitação vocal

**DOI:** 10.1590/2317-1782/20212021241pt

**Authors:** Mara Behlau, Anna Alice Almeida, Geová Amorim, Patrícia Balata, Sávio Bastos, Mauriceia Cassol, Ana Carolina Constantini, Claudia Eckley, Marina Englert, Ana Cristina Cortes Gama, Ingrid Gielow, Bruno Guimarães, Livia Ribeiro Lima, Leonardo Lopes, Glaucya Madazio, Felipe Moreti, Vanessa Mouffron, Katia Nemr, Priscila Oliveira, Marina Padovani, Vanessa Veis Ribeiro, Kelly Silverio, Thays Vaiano, Rosiane Yamasaki

**Affiliations:** 1 Centro de Estudos da Voz – CEV - São Paulo (SP), Brasil.; 2 Escola Paulista de Medicina, Universidade Federal de São Paulo – UNIFESP - São Paulo (SP), Brasil.; 3 Universidade Federal da Paraíba – UFPB - João Pessoa (PB), Brasil.; 4 Universidade Federal de Alagoas – UFAL - Maceió (AL), Brasil.; 5 Apta Comunicação, Recife (PE), Brasil.; 6 Universidade Federal de Pernambuco – UFPE - Recife (PE), Brasil.; 7 Centro de Fotobiomodulação e Saúde – CFOTOBIOS - Belém (PA), Brasil.; 8 Universidade Federal de Ciências da Saúde de Porto Alegre – UFCSPA - Porto Alegre (RS), Brasil.; 9 Universidade Estadual de Campinas – UNICAMP - Campinas (SP), Brasil.; 10 Faculdade de Ciências Médicas da Santa Casa de São Paulo – FCMSCSP - São Paulo (SP), Brasil.; 11 Universidade Federal de Minas Gerais – UFMG - Belo Horizonte (MG), Brasil.; 12 Clínica Bruno Guimarães Serviços de Fonoaudiologia e Fisioterapia, Fortaleza (CE), Brasil.; 13 Centro Universitário da Faculdade de Medicina do ABC – FMABC - Santo André (SP), Brasil.; 14 Complexo Hospitalar Municipal de São Bernardo do Campo – CHMSBC - São Bernardo do Campo (SP), Brasil.; 15 Universidade de São Paulo – USP - São Paulo (SP), Brasil.; 16 Faculdade de Odontologia de Bauru, Universidade de São Paulo – USP - Bauru (SP), Brasil.

**Keywords:** Voz, Distúrbio da Voz, Treinamento da Voz, Fonoterapia, Reabilitação Vocal

## Abstract

O presente texto é a continuação da publicação referente ao XVIII Congresso da SBFa. Na parte “A” apresentamos análises sobre avaliação clínica vocal. O foco da parte “B” são aspectos de reabilitação vocal: 4. Técnicas tradicionais de terapia vocal; 5. Técnicas modernas de eletroestimulação e fotobiomodulação aplicadas à reabilitação. Os inúmeros estudos sobre os diversos programas, métodos ou técnicas tradicionais de reabilitação, muitos de elevada qualidade de evidência, permitem considerar tais procedimentos relativamente bem descritos, seguros e com efeitos conhecidos, dando conta do tratamento de diversos distúrbios vocais. As evidências científicas com as técnicas tradicionais são reconhecidas mundialmente. Novas frentes de evolução, como o uso da eletroestimulação ou fotobiomodulação em voz parecem ser promissoras como abordagens coadjuvantes. Há mais estudos sobre eletroestimulação em voz do que com fotobiomodulação, contudo, evidências científicas para essas duas técnicas modernas são ainda limitadas. Conhecimento e cautela são necessários para a aplicação de quaisquer técnicas.

## INTRODUÇÃO

A Sessão Científica da sala Mérito Mara Behlau, ocorrida no dia 14 de dezembro de 2020, como uma das atividades do XXVIII Congresso Brasileiro de Fonoaudiologia, organizado pela Sociedade Brasileira de Fonoaudiologia – SBFa foi consolidada em duas publicações. Na parte “A”^(1)^ do presente artigo foram apresentados os principais dados científicos, comparando-se com a realidade clínica brasileira acerca dos três primeiros aspectos debatidos, a saber: 1. Julgamento perceptivo-auditivo da qualidade vocal; 2. Análise acústica do sinal vocal; 3. Autoavaliação em voz. Nessa segunda publicação, parte “B”, são explorados os aspectos relacionados à reabilitação vocal, apresentados em dois tópicos: 4. Técnicas tradicionais de terapia vocal; 5. Técnicas modernas de eletroestimulação e fotobiomodulação aplicada à reabilitação vocal.

Compreende-se por reabilitação vocal ou fonoterapia para voz um processo dinâmico e não linear com a finalidade de se desenvolver um melhor equilíbrio das estruturas e processos que respondem pela funcionalidade vocal, reduzindo uma desvantagem vocal ou aprimorando as qualidades estéticas de uma emissão^(2)^. Historicamente é uma combinação de artes e ciência, sendo que os primeiros exercícios de voz vieram do ensino do canto. A partir da década de 1950 houve um reposicionamento filosófico da reabilitação vocal, com a busca de fundamentos científicos para as técnicas propostas, a partir dos trabalhos seminais de Emil Froeschels^(3,4)^. A evolução do conhecimento fez-se em diversos domínios, mas, de modo geral, as contribuições para os processos de reabilitação vocal envolvem três aspectos comumente presentes na terapia de voz: 1. Mudanças comportamentais, 2. Ajustes musculares e/ou 3. Questões de autoimagem vocal, distribuídos em doses variáveis de acordo com o caso^(2)^.

A reabilitação fonoaudiológica age sobre a funcionalidade vocal e boa parte dessa ação é decorrente das técnicas empregadas. No presente texto, o foco principal está exatamente nos métodos e técnicas de reabilitação vocal, por ocuparem um espaço central na reabilitação fonoaudiológica, já que permitem ao indivíduo experimentar novas formas de produzir a voz, com ajustes musculares mais eficientes, favorecendo a experiência de se produzir uma voz mais harmônica e com menor esforço, após alguns minutos de execução de exercícios selecionados. O processo de reabilitação vocal é didaticamente apresentado em dois componentes: 1. A terapia vocal direta, direcionada à técnica vocal propriamente dita; 2. A terapia vocal indireta, na qual são incluídas abordagens para lidar com aspectos cognitivos, de comportamento e do ambiente, tais como controle de estresse e enfrentamento do problema, técnicas de relaxamento, aconselhamento vocal e aspectos ambientais como microfone, acústica da sala, principalmente nas vozes profissionais. Apesar de reconhecermos a importância de ambos os aspectos, escolhemos a terapia vocal direta como tema de discussão para redução do *gap* entre a ciência e a clínica, pois quando se analisam as evidências científicas, as principais concentram-se nas abordagens diretas^(5)^, analisadas nessa publicação.

Toda a ciência aplicada e, de modo geral, a área da saúde, apresenta dois movimentos para o avanço do conhecimento: a comprovação das chamadas abordagens tradicionais, muitas vezes empregadas há tempo e desenvolvidas de modo intuitivo e a proposição de intervenções inovadoras, por vezes já empregadas em outros campos do conhecimento. Os avanços tecnológicos e de pesquisa científica vem modificando a rotina clínica do fonoaudiólogo, seja no que diz respeito à avaliação do caso, como destacado na parte “A” desta contribuição^(6)^, mas também na administração de procedimentos de terapia. A partir dessa afirmação, apresentamos os principais dados das técnicas tradicionais de reabilitação vocal, seguidos de consideração sobre duas abordagens recentemente sugeridas como adjuvantes e empregadas na clínica vocal, a eletroestimulação e a fotobiomodulação.

## TÉCNICAS VOCAIS TRADICIONAIS NA REABILITAÇÃO VOCAL

É um desafio para o especialista em voz aplicar um tratamento eficiente utilizando as melhores evidências científicas disponíveis. Aliar os resultados da pesquisa científica com a decisão clínica envolve vários caminhos que vão desde a análise e interpretação dos resultados de uma pesquisa científica, até a formulação de evidências e diretrizes da prática clínica^(7)^. Como exposto na parte “A” dessa contribuição^(6)^, abordagens como a prática baseada em evidências^(8)^ incorporam o uso consciente e ponderado da melhor evidência de pesquisa na tomada das decisões clínicas. Isso pode parecer limitar as opções disponíveis à utilização apenas de técnicas e métodos comprovados e, embora essa pareça ser a melhor atitude, muitas vezes a clínica exige o uso de procedimentos ainda sem evidência. Nesses casos, a transparência na relação com o paciente e a busca de, pelo menos, evidências clínicas, é essencial.

Ancorado no exposto, apresentamos as principais evidências científicas e as decisões clínicas dos métodos e técnicas de reabilitação vocal mais pesquisados na área de voz, a saber: Método Lee Silverman, LSVT^®^, Método de Exercícios de Função Vocal (EFV); Método de Ressonância, Programa Integral De Reabilitação Vocal - PIRV; Método de Acentuação; Terapia Vocal para Idosos – TVI; Método de Terapia de Resistência Fonatória – PhoRTE; Método Proprioceptivo-Elástico – PROEL; Técnica de Massagem Laríngea; Técnica de Exercícios de Trato Vocal Semiocluído – ETVSO e Técnica do Som Basal.

O **Método Lee Silverman, LSVT^®^** apresenta como conceitos essenciais o foco exclusivo no aumento da intensidade da voz, com múltiplas repetições em tempo máximo de fonação e com variação de frequência da voz; é aplicado de forma intensiva, com quatro sessões por semana durante quatro semanas, totalizando 16 sessões^(9-11)^. Seus resultados estão ancorados em uma revisão sistemática da literatura^(12)^, com metanálise de nove ensaios clínicos randomizados^(9-11,13-17)^ que evidencia este método como um tratamento que oferece melhora da comunicação de pacientes com doença de Parkinson idiopática, tanto no formato de teleatendimento quanto no formato presencial, promovendo melhora da intensidade vocal e impactos positivos na qualidade de vida. Esse método é o mais estudado na reabilitação vocal, com o mais alto nível de evidência científica comprovada, sendo amplamente utilizado em diversos lugares do mundo; contudo, exige certificação específica para sua administração.

O **Método de Exercícios de Função Vocal - EFV)** é uma abordagem fisiológica de tratamento de distúrbios da voz baseado no desenvolvimento do equilíbrio fisiológico entre os subsistemas de respiração, fonação e ressonância, para fortalecer a musculatura intrínseca da laringe e ampliar a ressonância do trato vocal^(18)^. Uma revisão sistemática contendo 21 pesquisas clínicas^(19)^, sendo oito ensaios clínicos randomizados^(18,20-26)^ apresenta um nível de evidência de moderado a forte para o EFV como um método de tratamento eficiente para melhorar a função vocal de indivíduos com diferentes quadros de disfonias, presbifonia e para profissionais de voz^(19)^.

O **Método de Ressonância** recebe diferentes denominações e pode ser considerado como método ou técnica vocal: 1. Método: Terapia de Voz Ressonante^(27)^, Terapia de Voz Ressonante Lessac-Madsen - LMRVT^(28)^, Terapia de Ressonância^(29)^; e, 2.Técnicas: *Humming* ^(30)^, Técnica de Sons Nasais^(31)^ e Y-Buzz^(32)^. No Brasil, é mais utilizado como técnica vocal^(31)^. Sua aplicação, tanto como método ou como técnica vocal, visa a melhora da produção vocal, minimizando o impacto entre as pregas vocais e, consequentemente, lesões por fonotrauma^(27,33)^. De acordo com uma revisão sistemática que incluiu nove pesquisas clínicas^(34)^, sendo dois ensaios clínicos randomizados^(27,33)^, esse método apresenta nível de evidência moderada para quadros de disfonias comportamentais, com melhora da qualidade da voz e da eficiência fonatória^(34)^.

O **Programa Integral de Reabilitação Vocal - PIRV** é um método de reabilitação vocal proposto por um grupo brasileiro^(35)^, que privilegia cinco aspectos: corpo-voz, fonte glótica, ressonância, coordenação pneumofonoarticulatória e atitude comunicativa. Os resultados de um estudo clínico randomizado^(26)^ evidenciam a efetividade do PIRV, que promove resultados positivos na qualidade vocal, na função laríngea e na melhora da qualidade de vida de pacientes com disfonia comportamental.

O principal objetivo do **Método de Acentuação** é obter um melhor controle da produção da fala e da voz, por meio de um adequado suporte pulmonar associado a emissões rítmicas de vogais e de frases e a movimentos corporais, que restauram a simetria de vibração das pregas vocais e reduzem o esforço muscular glótico^(36)^. Sua abordagem holística foi comprovada por meio de um ensaio clínico randomizado^(37)^ que evidenciou melhora da qualidade vocal em quadros de disfonias hiper e hipofuncionais.

O programa **Terapia Vocal para Idosos – TVI** foi desenvolvido no Brasil para tratar, especificamente, quadros de presbifonia^(38)^. Os resultados do ensaio clínico randomizado^(38)^ comprovam a eficácia do TVI no tratamento vocal de idosos, promovendo melhoras na qualidade de vida e na qualidade da voz imediatamente após o tratamento e seguimento de um mês. Os benefícios obtidos pelo TVI no formato intensivo ou convencional são similares, diferenciando-se apenas na diminuição do arqueamento das pregas vocais que foi mais evidente no formato intensivo^(38)^.

O **Método de Terapia de Resistência Fonatória - PhoRTE**, é um método de reabilitação vocal para idosos com presbifonia^(23)^ adaptado do LSVT®^(9)^ e que tem como foco o aumento da intensidade vocal e da projeção de voz como um todo. Um ensaio clínico randomizado^(23)^ evidenciou que o PhoRTE melhora a qualidade de vida e a autopercepção de esforço fonatório de indivíduos presbifônicos.

O **Método Proprioceptivo-Elástico - PROEL** é um método italiano holístico de reabilitação vocal para quadros de disfonias funcionais e orgânicas^(39)^. Esse método tem como objetivo reequilibrar o sistema fonatório, eliminando a tensão muscular e buscando uma maior elasticidade no corpo. Uma menor tensão muscular no corpo produz uma maior elasticidade do sistema fonatório, levando a um estado ecológico máximo. Pesquisas com delineamento do tipo intervenção antes-após evidenciou melhora da qualidade vocal de pacientes com disfonia funcional^(39)^ e um estudo quasi-experimental a melhora da qualidade da voz de pacientes submetidos a laringectomia parcial horizontal^(40)^.

A **Técnica de Massagem Laríngea** é uma das opções da clínica vocal que apresenta maior nível de evidência científica. Há variações em sua aplicação, que pode ser feita com manipulação intensa via região anterior do pescoço e vocalizações associadas^(41)^ ou por acesso posterior, com ação suave de ambas as mãos, sem emissão vocal durante a manipulação^(42)^. Uma revisão sistemática^(43)^ que analisou dois ensaios clínicos randomizados^(44,45)^ e duas pesquisas do tipo intervenção antes-após^(46,47)^, indicaram a eficiência dessa abordagem aplicada nas disfonias comportamentais, por meio do equilíbrio muscular adquirido com o relaxamento da musculatura paralaríngea e a flexibilização da movimentação vertical da laringe, com diminuição da intensidade da dor corporal.

A **Técnica de Exercícios de Trato Vocal Semiocluído - ETVSO** inclui uma ampla variação de exercícios com vogais fechadas, sons fricativos sonoros, vibração de língua, lábios ou linguolabial, além de uma vibratórios até exercícios com diversas extensões artificiais do trato vocal, ou seja, tubos de ressonância, flexíveis ou rígidos, canudos largos e estreitos, para emissão sonorizada no ar ou na água. Considerando primeiramente os ETVSO que utilizaram tubos ou canudos, a pesquisa científica de maior relevância é a revisão sistemático de ETVSO na voz cantada^(48)^, que analisou seis artigos elegíveis^(49-55)^. De acordo com esse estudo^(48)^, exercícios com tubo de ressonância imerso em água, tubo flexível e canudos promovem efeitos positivos na qualidade vocal de cantores, com emissão mais confortável, mais projetada e mais econômica. Outras diversas contribuições^(51,56-60)^, incluindo dois ensaios clínicos randomizados^(61,62)^ mostraram que os ETVSO podem ser indicados para casos de hiperfunção vocal, disfonias comportamentais e também para o condicionamento vocal, auxiliando na sintonia fonte-filtro e adução glótica mais eficiente. Já para as técnicas dos sons vibrantes, considerados sons facilitadores, a maior evidência é obtida em dois ensaios clínicos randomizados^(62,63)^. A aplicação clínica desta técnica produz diversos efeitos e visa uma emissão vocal equilibrada e confortável, melhor fechamento glótico e maior quantidade de harmônicos. Outra técnica que faz parte dos ETVSO é a firmeza glótica; embora alguns estudos tenham sido realizados com esta técnica, principalmente comparando-a com outros ETVSO^(50,58,64)^, apenas um ensaio clínico randomizado^(65)^ foi publicado. A firmeza glótica promove melhor adução glótica, aumento da amplitude da vibração da mucosa e ampliação do trato vocal.

O nível de evidência da **Técnica do Som Basal** é de ensaio clínico randomizado. O som basal é um som facilitador emitido com maior contração do músculo tireoaritenóideo, capaz de gerar a desativação do ajuste muscular habitual, estimular movimento mucondulatório e favorecer a adução glótica. Embora de execução relativamente difícil, por se exigir a produção de um som não usual na comunicação, seu efeito comumente ocorre produzindo melhor qualidade vocal e maior facilidade na emissão.

Com todos esses resultados e evidências, a decisão do fonoaudiólogo especialista em voz na escolha do melhor procedimento, método ou técnica deve ser balizada em dois eixos principais: 1. No que se refere à pesquisa, a pergunta que precisa ser respondida é: como elaborar o melhor delineamento da pesquisa para desenvolver conhecimento científico aplicado à reabilitação fonoaudiológica?; 2. no eixo da clínica vocal – qual procedimento clínico apresenta as maiores evidências da pesquisa científica? Há evidências importantes nos métodos tradicionais de reabilitação vocal, alguns com propostas mais holísticas e outros mais específicos para certos quadros vocais. Faltam evidências do emprego da reabilitação vocal nas disfonias pediátricas, embora se reconheça a possibilidade de riscos vocais no longo-prazo, principalmente em meninas^(66)^.

## TÉCNICAS MODERNAS DE ELETROESTIMULAÇÃO E FOTOBIOMODULAÇÃO APLICADAS À REABILITAÇÃO VOCAL

### Eletroestimulação na reabilitação vocal

Diversas correntes elétricas podem ser usadas como recurso terapêutico com diferentes objetivos, seja para o manejo da dor, progresso na vascularização, cicatrização dos tecidos, ativação de metabolismo celular, melhoria da propriocepção, relaxamento, drenagem ou contração muscular. Dentre as inúmeras correntes existentes, as mais usadas na área de voz são a TENS (Estimulação Elétrica Nervosa Transcutânea) e a FES (Estimulação Elétrica Funcional); na literatura internacional, essa última corrente é, por vezes, denominada de NMES (Estimulação Elétrica Neuromuscular), mas há várias discussões a respeito devido à nomenclatura confusa trazida da área em que apoiamos nossos conhecimentos que é a Fisioterapia.

Os parâmetros fundamentais em eletroterapia e que definem o tipo da corrente elétrica são frequência, duração de pulso e intensidade, além da forma de onda. A combinação destes quatro parâmetros vai definir o tipo de corrente e também vários tipos diferentes de correntes e, portanto, provocar efeitos diversos nos tecidos.

Especificamente, em relação à TENS, os profissionais da clínica fonoaudiológica e os pesquisadores na área de eletroestimulação concordam e percebem que há confusões quanto ao nome desta corrente e suas variações. O nome TENS não é suficiente para evidenciar a sua combinação de parâmetros. Assim, seria mais indicado identificar o procedimento com uso do primeiro nome “TENS” acrescido do segundo nome, como por exemplo: “TENS ACUPUNTURA - em modulação de baixa frequência”; “TENS CONVENCIONAL – em modulação de alta frequência”; “TENS BREVE INTENSO”; “TENS BURST”; “TENS VIF - Variação de intensidade e frequência”.

A eletroestimulação na área de voz tem sido usada no Brasil desde 1986, aplicada a casos de paralisia laríngea na clínica vocal, usando-se a TENS BURST, com a finalidade de estimular contrações musculares na laringe. Logo em seguida, passou-se a aplicar a TENS em disfonias comportamentais, usando-se a TENS BREVE INTENSA, de alta frequência, com o objetivo de promover o relaxamento laríngeo. Com o avanço do conhecimento, passou-se a aplicar a TENS CONVENCIONAL de alta frequência para os casos de disfonia acompanhada de tensão muscular aguda e a TENS ACUPUNTURA de baixa frequência para os casos de disfonia com tensão muscular crônica ou dor laríngea crônica. A primeira publicação brasileira sobre eletroestimulação^(67)^ foi um relato de caso de paciente com disfonia espástica em adução, no qual aplicou-se TENS associada a exercícios de respiração e fonação, com a finalidade de alcançar relaxamento muscular, obtendo-se resultados positivos na voz e laringe até 60 dias após o início do tratamento. Contudo, pesquisas com eletroestimulação no Brasil, iniciaram somente em 2008, com um grupo de pesquisadores com fisioterapeutas e fonoaudiólogo aplicando-se TENS Acupuntura de baixa frequência em mulheres com disfonia comportamental^(68)^. Portanto, há ainda muito a ser investigado e comprovado nessa área.

Tratando-se da disfonia comportamental, alguns estudos foram realizados demonstrando duas formas diferentes de aplicação da TENS. A “TENS Acupuntura - em modulação de baixa frequência”, mas com alta intensidade no limiar motor^(45,68-72)^, em que dois eletrodos de um mesmo canal do aparelho gerador de corrente são distribuídos de forma ipsilateral: um fixado na região do músculo trapézio – fibras descendentes e o outro na região submandibular, (e repete-se a mesma distribuição do lado oposto), com campo de estimulação mais amplo, mas com estimulação menos profunda. Nesta configuração de corrente, o indivíduo recebe o estímulo em decúbito dorsal e não há emissão ou qualquer exercício de voz associado à corrente, durante a eletroestimulação, porque a estimulação recebida é forte, provocando um forte abalo na laringe. A outra forma de corrente pesquisada e muito difundida na clínica vocal é “TENS Acupuntura - em modulação de baixa frequência”, mas com baixa intensidade, no limiar sensório-motor^(73-77)^. Neste tipo de estimulação, dois eletrodos de um mesmo canal do aparelho gerador de corrente são colocados bilateralmente na lâmina da cartilagem tireóidea, estimulando, portanto, um campo menor, em que a estimulação é mais concentrada e mais profunda na região da laringe. Neste tipo de corrente recomenda-se a associação de exercícios vocais e a intensidade da eletroestimulação é leve.

A experiência clínica e as pesquisas concordam e demonstram que ambas as formas de TENS promovem analgesia, diminuição do esforço à fonação, levam ao equilíbrio muscular, com melhora dos sintomas laríngeos e vocais, bem como melhora da qualidade vocal, especialmente quanto à tensão vocal^(45,69-72,74-78)^. Tais efeitos foram verificados comparando-se os momentos antes e após intervenção com eletroestimulação, por meio de protocolos de autoavaliação, julgamento auditivo e avaliação acústica, fosse em estudo de efeito imediato como em intervenção por 12 sessões.

Embora a clínica e a pesquisa tenham vários pontos convergentes, há ainda pouca evidência científica sobre o assunto eletroestimulação na reabilitação de pacientes com disfonia. A literatura registra 13 estudos, com diferentes níveis de evidência, sendo dois de revisão sistemática^(78,79)^, sete ensaios clínicos randomizados^(69-71,74,78,79)^ três estudos quase-experimentais^(75,76,80)^ e um estudo de efeito pré-pós^(68)^. A maioria dos estudos contempla as disfonias comportamentais e essa é a maior contribuição das pesquisas brasileiras. Entretanto, destacam-se alguns pontos frágeis que merecem atenção: tempo longo entre finalização da pesquisa e publicação; amostras pequenas; diagnóstico laríngeo como critério de inclusão e a presença de outros sinais musculares e vocais que tornam a amostra menos homogênea; dificuldade de realizar avaliação mais objetiva da imagem laríngea e a dificuldade de seguimento dos casos tratados.

Por outro lado, a experiência clínica dos colegas que têm utilizado tais procedimentos, revela que o trabalho com a “TENS Acupuntura - em modulação de baixa frequência”, com baixa intensidade no limiar sensoriomotor, com campo de estimulação na laringe, pode regular a força muscular, funcionando como *biofeedback* quanto à intensidade vocal usada durante o exercício de voz, melhorando a propriocepção, levando ao equilíbrio laríngeo mais rapidamente. Entretanto, a clínica apresenta alguns pontos frágeis, reconhecidos pelos próprios especialistas, como a dificuldade de mostrar seus resultados na literatura, assim como a de documentar adequadamente os casos tratados, aplicar os conhecimentos em número maior de pacientes. Desta forma, a tomada de decisão nem sempre é baseada em evidências científicas na área, pois considera-se muito a experiência clínica, já que as pesquisas não trazem resultados tão rápidos na literatura para o consumo dos fonoaudiólogos clínicos.

Alguns pontos em que pesquisa e clínica tem dificuldades em convergir é sobre o controle necessário nas pesquisas científicas em relação ao ambiente, amostra, aplicabilidade da intervenção e desfechos a serem avaliados necessariamente iguais para validar os resultados, o que não é passível de ser implementado nos atendimentos clínicos. Para estudos científicos, a corrente aplicada nas intervenções com eletroestimulação precisa seguir igual, mesmo que as necessidades e características musculares dos indivíduos sejam diferentes. Por outro lado, a eletroestimulação na clínica é dinâmica e customizada para cada paciente, com autonomia do clínico na escolha dos parâmetros da corrente a ser aplicada.

As formas de correntes possuem indicações e fins específicos a serem considerados na hora da prescrição. A eficácia da eletroestimulação depende muito mais do conhecimento/competência profissional na tomada de decisão e aplicação da técnica em si. Entretanto, pesquisadores e clínicos concordam que há necessidade de mais estudos com nível mais elevado de evidência científica e também para responder aos diversos questionamentos que a prática clínica aponta. É importante considerar pesquisas futuras com FES, AUSSI e RUSSA, tanto nas disfonias não somente comportamentais, mas nas orgânicas também. Há pesquisas com FES, porém, o nível de evidência é muito baixo^(81-83)^.

Frente ao exposto, clínicos e pesquisadores concordam que a TENS pode ser aplicada isoladamente em casos de disfonia acompanhada de tensão e dor muscular. Caso seja aplicada a TENS ACUPUNTURA na região cervical, com intensidade forte no limiar motor, não se deve associar exercícios ou qualquer tipo de fonação. Ao se escolher a TENS CONVENCIONAL, com eletrodos na região laríngea e estímulos leves, em que a tensão muscular não é acompanhada de dor, pode-se aplicar a corrente associada às técnicas vocais tradicionais. Ambas as formas de aplicação são consideradas coadjuvantes no processo terapêutico.

As medidas multiparamétricas talvez auxiliem a compreender melhor os resultados das intervenções com eletroestimulação, assim como avaliações que envolvem dosimetria. Considera-se que a interação entre a pesquisa e a clínica sejam aspectos fundamentais a serem ponderados na resolução das questões trazidas por ambas para o avanço científico na área de voz.

### Fotobiomodulação na reabilitação vocal

A terapia por fotobiomodulação consiste em uma modalidade terapêutica na qual a irradiação de luz no espectro visível e/ou infravermelho próximo promove mudanças biofísicas e bioquímicas no organismo, resultando em respostas biológicas como modulação da inflamação, redução de edema, de dor e melhora da regeneração tecidual^(84)^. Esse tipo de terapia é conhecido por mais de cinco décadas, mas ainda não obteve ampla aceitação principalmente pelos mecanismos de ação molecular, celular e tecidual não serem bem aceitos^(85)^.

Dentre as diversas fontes de luz que podem ser utilizadas para esse fim, na Fonoaudiologia destacam-se o LASER (*Light Amplification by Stimulated Emission of Irradiation*) de baixa potência e o LED (*Light Emitting Diodes*), sobretudo os comprimentos de onda na faixa do vermelho (635–700 nanômetros) e infravermelho próximo (808-1100 nanômetros). O efeito do LASER usado na terapia por fotobiomodulação não é térmico e nem ablativo. Por esse motivo, os equipamentos utilizados são aqueles registrados na Agência Nacional de Vigilância Sanitária - ANVISA, o que dá segurança ao clínico por terem sido verificados e aprovados. O LASER usado na clínica vocal é de baixa potência, sem efeitos bionegativos sobre o tecido biológico, atuando sobretudo no estímulo à fisiologia do organismo. Os efeitos terapêuticos provenientes da fototerapia decorrem da absorção de fótons pelos cromóforos, que são conjuntos de átomos ou partes de uma molécula capazes de absorver luz na região do ultravioleta ou do espectro visível, sendo também responsáveis pela cor de uma molécula^(86)^.

Embora os mecanismos celulares associados à fotobiomodulação ainda não sejam completamente compreendidos^(85)^, a teoria mais aceita até o momento postula que a absorção do vermelho e do infravermelho ocorra na mitocôndria, mais precisamente na cadeia respiratória, favorecendo a produção de adenosina trifosfato (ATP), a regulação do pH intracelular, a ativação de mecanismos antioxidantes e a recuperação da homeostase celular^(87,88)^.

O uso desses recursos terapêuticos na área de voz ainda é um campo muito pouco explorado e, embora tenha se popularizado na última década, tem como base as evidências advindas de outras especialidades na saúde, como a fisioterapia, odontologia, cardiologia e medicina esportiva. Em quadros de edema e inflamação, por exemplo, a terapia luminosa melhora a microcirculação local, a angiogênese, inibe mediadores químicos e ativa células de defesa e enzimas antioxidantes^(89)^. Na regeneração tecidual, por sua vez, as evidências demonstram eficácia tanto em lesões superficiais como também em tecidos mais profundos^(90)^, mostrando-se eficiente na proliferação de fibroblastos, na síntese e organização de colágeno e na indução de neovascularização^(91,92)^. Na medicina esportiva os efeitos da fotobiomodulação sobre o sistema muscular são cada vez mais robustos, com destaque para o aumento da resistência à fadiga, da força e da velocidade de recuperação dos atletas^(93-96)^ bem como sua eficácia no relaxamento cervical e alívio da dor muscular^(97)^.

De modo geral, efeitos como redução de edema, modulação da inflamação, melhora do desempenho/relaxamento muscular e recuperação tecidual foram analisados em uma revisão sistemática que mostrou resultados positivos em 13 de 16 estudos, apesar da heterogeneidade e de questões metodológicas^(95)^. A aplicação de terapia de fotobiomodulação associada a exercícios em indivíduos saudáveis mostrou melhoria na performance muscular e redução de fadiga em uma recente revisão sistemática com metanálise, comparando os resultados de 28 estudos em relação a placebo^(95)^. Finalmente, uma revisão integrativa mostrou que brasileiros têm se destacado em publicações sobre a fotobiomodulação na área da saúde e, de modo geral, o LASER de baixa intensidade age sobre o desempenho muscular, reduzindo fadiga, aumentando força, resistência muscular e melhorando marcadores químicos; a diversidade metodológica dos estudos dificultou a identificação de parâmetros eficazes para a obtenção de resultados^(98)^.

Na ciência vocal, um estudo pioneiro sobre o uso da fotobiomodulação para tratamento da laringite por refluxo laringofaríngeo induzido por intubação em ratos mostrou que o laser beneficiou o reparo tecidual da laringe nos animais irradiados quando comparados aos não irradiados. Segundo os autores, os resultados indicam que o uso desse recurso pode ser benéfico para a população com distúrbios da voz, inclusive para aqueles com alterações teciduais na lâmina própria^(99)^. O LED foi testado como uma possibilidade de atenuação dos sintomas de fadiga vocal^(100)^, a luz vermelha foi eficaz para melhorar marcadores relacionados à acústica, aerodinâmica e à autopercepção de indivíduos saudáveis submetidos a uma tarefa de esforço vocal. Os achados indicam que a fotobiomodulação pode ser um recurso promissor para a redução da fadiga vocal. Deve-se determinar os parâmetros dosimétricos ideais, a combinação ou não da fotobiomodulação com exercícios vocais e o momento ideal de aplicação. Finalmente, o uso da fotobiomodulação em pregas vocais humanas, *in vitro* ^(101)^ e *in vivo* ^(102)^ em pregas vocais de coelhos, revelou que o comprimento de onda vermelho aumentou a proliferação e migração de células do epitélio de prega vocal, apontando uma possibilidade futura do uso desse recurso na cicatrização de lesões laríngeas.

Apesar dos dados disponíveis serem ainda preliminares e recentes, a fototerapia é um tratamento simples que pode trazer benefícios para a área de voz, sobretudo em indivíduos saudáveis com foco no treinamento muscular. No entanto, seu uso deve ser controlado por profissionais habilitados e que sejam capazes de aplicar doses adequadas, no momento adequado, na localização correta para que se alcance o efeito desejado. Do mesmo modo que observado na eletroestimulação, a aplicação de fotobiomodulação deve ser embasada em um completo conhecimento das condições clínicas gerais e locais da aplicação, com atenção especial para a região da cabeça e pescoço.

## COMENTÁRIOS

A literatura apresenta muitos estudos na área da voz com as mais variadas técnicas vocais, com delineamentos e níveis de evidência diversos. A pesquisa na área de voz tem avançado e já temos técnicas estudadas em estudos clínicos randomizados e revisões sistemáticas. Muita atenção tem sido dada na compreensão dos efeitos das técnicas tradicionais da terapia de voz e podemos dizer que a década passada foi a década de estudo relacionados à comprovação dos efeitos da terapia de voz^(6)^. Os resultados das pesquisas científicas permitem reconhecer que as abordagens tradicionais dão conta da variedade de casos vocais, são seguras, apresentam passos relativamente definidos, sendo muitas delas usadas mundialmente, como o Método de Ressonância^(27-34)^. Reconhece-se ainda que as abordagens tradicionais de reabilitação vocal produzem efeito não somente sobre a voz, mas sobre a competência comunicativa e aspectos emocionais dos pacientes^(6)^. Tanto métodos específicos, como o LSVT, para indivíduos com Doença de Parkinson^(9-11)^, como holísticos, como o PROEL^(39,40)^ produzem efeitos positivos imediatos e no curto-prazo. A maior concentração de evidências de qualidade sobre os efeitos da intervenção fonoaudiológica concentram-se sobre as abordagens tradicionais de reabilitação.

Um levantamento de toda a coleção do *Journal of Voice - JoV* posiciona o Brasil como o segundo país que mais contribuiu para publicações sobre terapia, perdendo apenas para os Estados Unidos da América^(6)^. Essa análise das publicações do JoV ao longo das décadas, mostrou claramente a evolução dos fonoaudiólogos como pesquisadores, por meio de estudos com escrita cientifica e melhor definição de termos, melhor delineamento metodológico, definição mais clara de variáveis, seleção adequada de desfechos, melhor descrição dos participantes e efeitos positivos, de grau variável, das técnicas, métodos e programas de terapia. É interessante comentar que foram identificadas 49 diferentes abordagens^(6)^, utilizadas de modo isolado ou combinado, o que permite considerar que os métodos, técnicas ou programas abordagens tenham alguns componentes ativos comuns, que deveriam ser mais bem compreendidos em uma proposta de taxonomia para terapia de voz. Recentemente, uma proposta desta natureza^(103)^ agrupou os instrumentos de intervenção direta em 5 categorias, de acordo com o subsistema primariamente envolvido na execução dos exercícios, a saber: 1. Auditiva; 2. Função vocal; 3. Musculoesquelética; 4. Respiratória; e 5. Somatossensorial. Essa taxonomia de programas terapêuticos utilizando-se uma terminologia padrão e uma estrutura para ajudar a pesquisa sistemática tem como objetivo reduzir o fenômeno da “caixa preta” na terapia de voz, que dificulta compreender os ingredientes ativos de um programa de reabilitação, prejudicando o desenvolvimento clínico e científico da especialidade.

Quanto às técnicas modernas recentemente utilizadas na terapia de voz, a eletroestimulação tem se apresentado como uma opção terapêutica interessante quando associada às técnicas tradicionais nos quadros comportamentais, com hiperfunção laríngea ou em condições de hipofunção, de causas neurológicas ou funcionais, demandando raciocínio clínico distinto na seleção deste recurso. Para ambas as indicações, vale ressaltar que é uma estratégia que, além de favorecer aumento da força muscular ou de auxiliar na redução de contrações musculares excessivas, pode favorecer a criação de um feedback que maximize o desempenho muscular, após exercícios de contração muscular voluntária^(104,105)^. Esta possibilidade pode facilitar a organização de novos engramas motores laríngeos, por fornecer feedback tátil-proprioceptivo, facilitando a repetição ou quebra de um padrão que precisa ser modificado.

Mais recentemente, tem-se discutido a eletroterapia como um importante agente modulador durante a execução da ação motora volitiva, influenciando o desempenho funcional^(106)^. Portanto, o uso deste recurso pode promover uma integração sensoriomotora, na qual as vias sensoriais são sensibilizadas pelo *feedback* periférico proprioceptivo provocado pela movimentação do segmento, ativando momentaneamente o córtex sensoriomotor^(107)^. Desta forma, enfoque na modulação sensorial pode também ser considerado, trazendo novas possibilidades a serem pesquisadas em condições de hiper ou hipossensibilidade, como tosse crônica refratária, por exemplo.

Estudos analisados em uma revisão integrativa^(108)^ apontam benefícios com a eletroestimulação na reabilitação de pacientes na clínica fonoaudiológica, porém, há diversidade metodológica, dificultando a comparação entre os achados. Sugere-se seleção de amostras mais homogêneas, com detalhada descrição metodológica da dosimetria utilizada e efeitos não somente imediatos. Os avanços que se observam para a utilização da eletroterapia associada às técnicas tradicionais de fonoterapia não prescindem da cuidadosa observação das contraindicações, especialmente pela região a ser aplicada, a cervical, ricamente vascularizada, com a presença do seio carotídeo, e inervada por parte dos nervos cranianos, além de outros aspectos, tais como uso de outros dispositivos elétricos e epilepsia, por exemplo.

Frente ao que foi exposto, é importante utilizar métodos de avaliação na rotina clínica e na pesquisa para verificar condições musculares dos pacientes buscando maior efetividade da aplicação da TENS. Pode ser utilizada uma escala analógica de dor musculoesquelética que avalia a região perilaríngea em repouso e durante a fonação, a *Laryngeal Palpatory Scale* ^(109)^. Uma recente revisão sistemática^(79)^ analisou 8 de100 artigos que preencheram os critérios de inclusão, de dois laboratórios respeitados (Brasil e Iran) com 87,5% dos estudos mostrando resultados efetivos após intervenção, com redução de dor nas áreas da laringe, pescoço, ombros, costas e masseteres. Os estudos também apontaram melhoria no julgamento perceptivos da qualidade vocal e redução nas sensações de dor, inchaço na garganta e força para falar. A literatura da fisioterapia, que já usa essa abordagem em muitas condições e há muito tempo, destaca a necessidade do conhecimento das características fisiológicas e metodológicas para sua aplicação, tanto em uma musculatura saudável quanto em uma musculatura alterada, para que se possa conduzir a otimização do uso da técnica de modo eficaz e com segurança na prática clínica e para fins de pesquisa^(110)^.

Ressalta-se a necessidade de cautela na aplicação da eletroestimulação e a necessidade de se conhecer a anatomia na região cervical para se garantir uma prática segura e responsável. A Fonoaudiologia tradicionalmente trabalha em equipe, com bom relacionamento com todos os membros da equipe de cuidados do indivíduo, que pode confirmar a aplicação de TENS, quando necessário. Essa prática responsável para a aplicação da TENS, ou de qualquer outra forma de eletroestimulação, é essencial para o profissional que se propõe a utilizar este recurso na região da cabeça e pescoço.

Finalmente, quanto à terapia moderna por fotobiomodulação, essa é uma técnica não invasiva aplicada por muitas disciplinas. Especificamente quanto às questões vocais, a fotobiomodulação aplicada à região laríngea tem como objetivo melhorar a qualidade vocal; contudo, não há ainda estudos científicos que comprovem a evidência positiva de sua utilização. Deve-se ressaltar que, apesar do seu potencial, o uso da fototerapia deve somente ser indicado como um recurso terapêutico adjuvante, informando ao cliente/paciente que seus efeitos ainda não são cientificamente comprovados na área de voz. No que diz respeito à técnica de aplicação, é fundamental proceder com adequada palpação da região laríngea e suas respectivas estruturas, a fim de obter segurança e precisão da área a ser irradiada.

Algumas contraindicações incluem: história de carcinoma e irradiação da região do pescoço^(111)^. A região cervical é altamente vascularizada, sendo fundamental o conhecimento de anatomofisiologia e do histórico do paciente antes de propor este procedimento, assim como das técnicas de aplicação e suas contraindicações. Deve-se ter o cuidado de uma avaliação diagnóstica médica prévia, com exames complementares, uma vez que quadros como hemorragias de prega vocal, neoplasias, papiloma ou leucoplasias requerem precaução no uso, sendo alguns deles contraindicações para o uso da fotobiomodulação. Atenção especial também deve ser dada à glândula tireóidea, localizada na região anterior do pescoço com limite superior no bordo inferior da cartilagem tireoide. Por não se conhecer ainda o efeito que este recurso pode provocar no tecido glandular, sugere-se investigar possíveis alterações tireoidianas, com maior destaque ao hipertireoidismo^(111)^. A investigação do uso de medicamentos fotossensíveis que podem causar manchas na pele do paciente com o uso da luz, também merece atenção.

A fotobiomodução também tem sido utilizada como tratamento em pacientes com doenças neurológicas, como Parkinson e sequelas de acidentes vasculares cerebrais, para melhora do metabolismo cerebral e regeneração neuronal ^(110)^, bem como em oncologia no tratamento da radiomucosite e xerostomia consequentes de rádio e quimioterapia. Neste sentido, também tem seu papel no tratamento de pacientes com alterações vocais e disfágicas associadas a estas doenças; no entanto, é necessária cautela no uso da fotobiomodulação, especialmente em pacientes oncológicos, devido ao efeito potencial de reativação de tumores^(112)^.

A fotobiomodulação, por si só, não é capaz de reabsorver ou eliminar lesões e também não influencia nos comportamentos prejudicais à voz. Sendo assim, é preponderante para a obtenção de bons resultados que o profissional priorize o raciocínio clínico e não um recurso terapêutico, de modo isolado.

Os dados apresentados mostram o dinamismo na área de voz e a busca de os estudos científicos se refletirem em prática clínica consciente. Os principais comentários dos seis aspectos da clínica vocal discutidos nas partes “A” e “B” do presente texto estão apresentados no Quadro 1.

**Quadro 1:** Pontos importantes dos seis aspectos da clínica vocal discutidos: julgamento perceptivo auditivo, avaliação acústica, autoavaliação em voz, técnicas vocais de terapia vocal, eletroestimulação laríngea e fotobiomodulação em voz

**Aspectos da clínica vocal**	**Pontos importantes**
Julgamento Perceptivo Auditivo	• O julgamento auditivo é considerado padrão-ouro na clínica vocal pelo fato de a voz ser um fenômeno perceptivo.
	• O julgamento profissional exige treinamento específico.
	• Confiabilidade variada de acordo com tipo de estímulo, tarefa, experiência dos avaliadores, entre outros.
	• Rugosidade, soprosidade e tensão são os três principais desvios vocais.
	• Escalas GRBASI e CAPE-V são as mais utilizadas.
	• GRBASI é mais simples, mas pouco sensível a mudanças menos evidentes na qualidade vocal.
	• CAPE-V exige mais treinamento, porém, identifica melhor os incrementos com o tratamento
Avaliação Acústica	• Documentação essencial na clínica vocal.
	• Qualidade do registro é crucial para sua confiabilidade.
	• Permite avaliação da contribuição da fonte e dos filtros.
	• Oferece informações sobre a produção vocal.
	• Programas de uso público ou de baixo custo são disponíveis.
	• Torna a voz mais concreta, permitindo associação viso-vocal.
	• Pode ser realizada de modo descritivo, independentemente do tipo de sinal vocal, pela análise espectrográfica.
	• Pode ser realizada por extração de parâmetros acústicos dependendo ou não da extração da fundamental.
	• Parâmetros isolados e que dependem da extração da frequência fundamental tem valor limitado.
	• Índices multiparamétricos ou medidas cepstrais são mais confiáveis e promissores.
	• Correlação moderada com o julgamento auditivo
Autoavaliação em Voz	• Contribuição inquestionável na avaliação vocal.
	• Explora a perspectiva única de quem vive com o problema de voz.
	• Oferece dados que não podem ser estimados por outras avaliações.
	• Protocolos desenvolvidos em outras línguas exigem adaptação cultural, linguística e validação com verificação das propriedades psicométricas para uso em português.
	• Em conjunto com o julgamento perceptivo-auditivo e a avaliação acústica forma uma tríade importante de avaliação vocal do indivíduo.
Técnicas Tradicionais de Terapia Vocal	• Há uma variedade de técnicas, métodos e programas analisados com estudos de diversos delineamentos e qualidade metodológica variável.
	• As maiores evidências de comprovação científica da terapia vocal foram produzidas com essas técnicas.
	• Programas como LSVT e EFV concentram o maior número de estudos.
	• Exercícios de trato vocal semiocluído são variados, descritos há muito tempo e submetidos e diversos estudos científicos.
	• O Brasil ocupa espaço importante no estudo de técnicas tradicionais de terapia vocal
Eletroestimulação em Voz	• Utilizada em outras ciências da saúde, tem mostrado resultados interessantes na clínica vocal.
	• Já produziu evidências de qualidade alta sobre seu efeito no tratamento de certas disfonias.
	• O tipo de corrente utilizada tem especial consideração na aplicação clínica.
	• Suas diferentes correntes e ajustes de parâmetros podem permitir enfoque laríngeo sensorial, motor ou ambos.
	• É vista como tratamento coadjuvante e não como recurso único nos casos de disfonia
Fotobiomodulação em Voz	• Utilizada em outras ciências da saúde, ainda precisa produzir evidências científicas na clínica vocal.
	• A escolha dos parâmetros dosimétricos tem especial consideração na aplicação clínica.
	• É vista como tratamento coadjuvante e não como recurso único nos casos de disfonia.
	• Pode ser utilizada também para condicionamento vocal

## CONCLUSÃO

A reabilitação vocal é responsabilidade do fonoaudiólogo especialista em voz, que trabalha para se recuperar a funcionalidade vocal e obter o melhor resultado possível de acordo com a condição anatomofunctional do paciente. As opções para se propor um programa de reabilitação são variadas. Escolher com sabedoria é uma atitude essencial para a melhoria dos serviços fonoaudiológicos e para uma percepção positiva dos outros profissionais da saúde, dos seguros de saúde e do público leigo. Exatamente essa afirmação, – escolher com sabedoria (*Chosing Wisely*), foi o lema de uma campanha criada pela *American Board of Internal Medicine* – ABIM, em 2012, para se reduzir a utilização de intervenções de baixo valor para o paciente, evitando-se testes ou práticas clínicas desnecessárias^(113)^. Essa campanha, lançada em 2012 hoje conta com a adesão de mais de 70 sociedades na área da saúde e mais de um milhão de clínicos parceiros da campanha. Evitar práticas sem comprovação é responsabilidade profissional e está muito presente em recomendações na neurologia, obstetrícia, terapia intensiva, geriatria, pediatria, enfermagem e fisioterapia, entre outras disciplinas.

Apesar de grande vitalidade clínica e científica, a área de voz ainda precisa avançar na pesquisa para a obtenção de melhores evidências. Evidências sobre as técnicas tradicionais de terapia são disponíveis na literatura, muitas delas produzidas por grupos brasileiros. Pesquisadores precisam ter melhor formação para propor desenhos de experimentos mais robustos. Clínicos necessitam de treinamento para consumir a ciência de forma consciente e crítica e proceder a uma tomada de decisão adequada. Vale lembrar que os resultados das pesquisas são, muitas vezes, interpretativos e obtidos em condições laboratoriais ideais. Todos esses aspectos devem ser levados em consideração. Reduzir o *gap* entre a ciência, por meio de análise de pesquisas, e a clínica vocal, que deve indicar quais problemas merecem ser investigados, deve ser uma responsabilidade compartilhada pelos profissionais de uma disciplina.

Escolher sabiamente é honrar as pesquisas científicas, necessário para o avanço clínico que tanto buscamos, além de refletir respeito ao paciente que nos procura. Uma formação acadêmica apoiada no melhor conhecimento disponível e uma prática consciente e compatível com a ciência são responsabilidades acadêmicas importantes de todas as instituições e clínicos que se propõem a ensinar os colegas.
